# A retrospective observational study of medical incident command and decision-making in the 2011 Oslo bombing

**DOI:** 10.1186/s12245-015-0052-9

**Published:** 2015-03-04

**Authors:** Rune Rimstad, Stephen JM Sollid

**Affiliations:** Department of Research and Development, Norwegian Air Ambulance Foundation, Holterveien 24, 1448 Drøbak, Norway; Medicine, Health and Development, Oslo University Hospital, Kirkeveien 166, 0424 Oslo, Nydalen Norway; Department of Industrial Economics, Risk Management and Planning, University of Stavanger, Kjell Arholms gate 41, 4036 Stavanger, Norway; Faculty of Social Sciences, University of Stavanger, Kjell Arholms gate 41, 4036 Stavanger, Norway; Air Ambulance Department, Oslo University Hospital, Kirkeveien 166, 0424 Oslo, Nydalen Norway

**Keywords:** Decision-making, Emergency medical services, Emergency medicine, Leadership, Mass casualty incidents, Observational study, Risk management

## Abstract

**Background:**

A core task for commanders in charge of an emergency response operation is to make decisions. The purposes of the study were to describe what critical decisions the ambulance commander and the medical commander make in a mass casualty incident response and to explore what the underlying conditions affecting decision-making are. The study was conducted in the context of the 2011 government district terrorist bombing in Norway.

**Methods:**

The study was a retrospective, descriptive observational study collecting data through participating observation, semi-structured interviews, and recordings of emergency medical services’ radio communications. Analysis was conducted using systematic text condensation. The ambulance commander was interviewed using the critical decision method.

**Results:**

The medical emergency response lasted 6.5 h, with little clinical activity after 2 h. Most critical decisions were made within the first 30 min, with the ambulance commander making the bulk of decisions. Situation assessment and underlying uncertainties strongly affected decision-making, but there was a mutual interaction between these three factors that developed throughout the different stages of the operation. Knowledge and experience were major determinants of how easily commanders picked up sensory cues and translated them into situation assessments. The number and magnitude of uncertainties were largest in the development stage, after most of the critical decisions had been made.

**Conclusions:**

In the studied mass casualty incident, the commanders made most critical decisions in the early stages of the emergency response when resources did not meet demand. Decisions were made under significant uncertainty and time pressure. Ambulance and medical commanders should be prepared to make situation assessments and decisions early and be ready to adjust as uncertainties are reduced.

**Electronic supplementary material:**

The online version of this article (doi:10.1186/s12245-015-0052-9) contains supplementary material, which is available to authorized users.

## Background

Commanders are the individuals appointed to be in charge of an emergency response operation. A core task for commanders is to make decisions [1-3]. Narratives and analyses of mass casualty incidents will typically not contain detailed descriptions of what decisions the commanders made. Based on a previously published case description of the 2011 Oslo bombing, the aim of this study was to probe deeper into the actions of the commanders to contribute to the empirical knowledge base on incident command and decision-making [4]. The research questions are more focused on the ‘what’ than the ‘how’: What critical decisions do the ambulance commander and the medical commander make in a mass casualty incident emergency response? What are the underlying conditions affecting decision-making?

In the Norwegian incident command system, a police incident commander provides overall command. The prehospital health resources are jointly commanded by an ambulance commander (emergency medical technician or paramedic), which appoint sub-commanders and organize the incident scene, and a medical commander (physician), which has the overall medical responsibility of triage, treatment, and evacuation and assign tasks to physicians and nurses on scene. The commanders’ role descriptions are on a national level outdated and under debate.

Analyses of how experienced commanders make decisions have been made in several fields: police, fire and rescue, high-risk industry, military, and emergency medical services [3,5-10]. The predominant paradigm in this research field in recent years is that of naturalistic decision-making. Characteristics of real-world decision settings include uncertain dynamic environments, time stress, high stakes, multiple players, and ill-structured problems [11]. Experienced practitioners do not usually develop and compare options but make rapid decisions of what actions to initiate based on recognition of familiar situations through critical cues [12]. Repeated situation assessment leads to later adjustments of the strategy.

Studies from the emergency medical services are underrepresented in the decision-making literature and even more so when it comes to prehospital commanders’ decisions in mass casualty incidents. A few articles focus on triage and triage decisions [13-15]. Some present information systems that could help commanders in specific decision situations or support pre-event planning [16,17].

**Figure 1:**
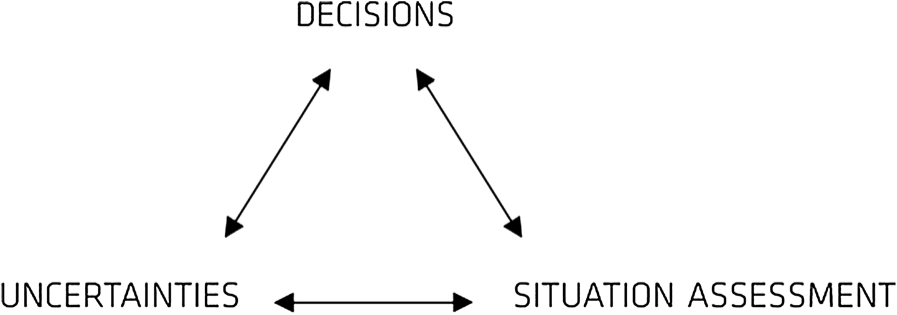
**Interactions between decisions, uncertainties, and situation assessment.** Situation assessment is the basis for decisions, and uncertainties influence decision-making. However, decisions affect how uncertainties are perceived and how the situation is assessed. Uncertainties also affect the situation assessment, and the importance of uncertainties depends on the assessment of the situation. There is, in other words, an interdependency between the three factors.

The following article presents how differences in experience and pre-event status affected how the ambulance commander and the medical commander interpreted their roles and executed their tasks. The timeline of the emergency response operation is divided in stages with differences in situation assessment that affect decision-making. Critical decisions and the underlying uncertainties are mapped in each stage. The final discussion explores the interaction between situational awareness, uncertainties, and critical decisions, cf. Figure 1.

## Methods

The study is a retrospective, descriptive observational study. Data was collected through participating observation, interviews, and recordings of radio communications.

Radio communications between the dispatch center, commanders, and ambulance crews on scene and elsewhere in the local area were routinely logged and stored as a collection of electronic audio files. This material contained confidential patient information. Permission to use the material without asking for consent from the involved patients was given by the Regional Ethics Committee (#2012/1673/REK), on the condition that personal and clinical information on single patients were omitted at transcription. The audio log files were then retrieved and transcribed with the due omissions.

Permission to conduct interviews with personnel involved in the incident response was given by the Ethics Committee at Oslo University Hospital (#2012-15778). Potential informants were identified from the radio communication transcripts. The ambulance commander, assisting ambulance commander, the four paramedics filling sub-commander roles on the incident scene, and two operators at the dispatch center were contacted by e-mail and asked to participate as informants in the study. The e-mail contained information about the aims of the study and job-related information about the interviewer. All personnel with some form of commander or sub-commander roles were invited to participate, and they all accepted the invitation and returned written consent. Some of the informants were known to the researchers as colleagues through several years. Others were unknown beforehand.

An interview guide was developed on basis of preliminary coding and analysis of the radio communication transcripts, cf. Additional file 1. Eight semi-structured interviews were conducted between May and June 2013 at the researcher or participant’s workplace, with no third party present. Six interviews lasted between 45 and 60 min, while one interview with a paramedic that was appointed a sub-commander but reassigned to other tasks and therefore had little direct contact with the studied commanders during the incident only lasted 25 min. The ambulance commander was interviewed last, using the critical decision method with several sweeps through the incident to (1) establish and verify a timeline, (2) deepen the understanding of decision-making processes, and (3) explore specific themes using so-called ‘what if’ queries [18]. This interview lasted 90 min. Interviews were audio recorded and transcribed. Brief field notes were taken. Demographic data were not collected. Each participant was only interviewed once. Participants were not asked to comment or correct transcripts or findings.

One researcher served as medical commander in the emergency response to the 2011 Oslo bombing, thereby conducting participating observation and earning familiarity with the specific case context before the research project was conceived. Field notes were taken 5 days after the incident and later expanded on basis of the later developed interview guide.

The total data material consisted of transcripts of radio communications, interviews, and field notes. These texts were analyzed using systematic text condensation as described by Malterud [19,20]. Its main structure can be summarized as four steps: (1) get an overview, (2) identify meaning-bearing units, (3) abstract the contents of each unit, and (4) summarize the implications [20]. Both researchers performed the first two steps independently and then the next steps together. Preliminary analysis was performed to develop the interview guide. The total text material was then analyzed as a whole. The coding tree (i.e., the categories used to structure meaning-bearing units) was developed during analysis as a central element. The final coding tree consisted of three main categories: situation assessment, decisions, and uncertainties. The research questions were rephrased in the final stages of analysis to more strongly highlight these categories as independent and interdependent units.

As presented by Burke, the identified critical decisions were classified as standard (taught explicitly or so common that everyone would agree as to the alternatives), typical (modifications to standard operating knowledge to meet the requirements of the situation), or constructed (no standard solution available; typically involve creative problem solving) [7]. Burke’s nomenclature of stages in an emergency response operation was also used [7].

The temporal extension of each described uncertainty and the amount of uncertainty at each point in time were evaluated by both researchers together based on the interviews, which specifically addressed these issues, and the amount of focus in radio transmissions. For illustrative purposes, each uncertainty was coded along the timeline as not present, small, or large.

## Results

In the afternoon of 22 July 2011, a single perpetrator detonated a car bomb in the government district of Oslo, Norway. He later involved in a shooting spree at a youth camp. The focus of this study was the ambulance and medical commanders’ decisions and actions on the government district scene. The emergency medical dispatch operators make decisions regarding mobilization and dispatch of resources, while decision-making and management on scene is the responsibility of the commanders. Dispatch center decisions have not been a subject of analysis.

The overall analysis revealed situation assessment (including situational awareness), uncertainties, and critical decisions as the major themes. Results from each theme is presented and illustrated in more detail. Their relationship and interdependencies are then discussed.

### Situation assessment

Situation assessment is based on sensory cues like sound, smell, and even taste, as well as what is visible. Cues are interpreted and compared to previous experiences, often on an unconscious level. This section starts with a description of the cues available to commanders on this particular incident ground, cf. Table 1. The impact of personal experience from large-scale incidents on situation assessment is highlighted, before illustrating how changes in situational awareness may be expressed by dividing the incident timeline into subsequent stages.

**Table 1:** **Cues to situation assessment**

**Type of cues**	**Cues**
Visual	Smoke
	People escaping the incident
	Casualties
	Broken windows
	Falling debris
	Fire
	Damaged facades
	Mobilized rescue vehicles
Olfactory	Burnt explosive material
	Smoke
Audio	Explosion
	Falling glass
	Fire alarms

Commanders use visual, olfactory, and audio cues to make situation assessments by matching previous experience and knowledge to distinct cues.

Before receiving calls to the dispatch center, nearby ambulances reported the explosion based on the loud sound, accompanying ground shaking from the shock wave, and a large smoke cloud. The amount of buildings with extensive façade damage gave an impression of the incident as huge. A less prominent cue was the smell of smoke from building fires.

Hundreds of people spontaneously evacuated the area around the explosion site. They looked distressed, anxious, or even aggressive, and a couple of them physically tried to drag ambulance personnel out of their cars to underpin how urgent help was needed.

Previous experience with actual mass casualty incidents was helpful in grasping or making sense of the situation. Among the informants were personnel with service experience from war-like situations and from relief work in natural disasters. A couple of informants had through many years paid a special interest in preparing for large-scale incidents and therefore taken courses and read up on books and articles on the subject. Those with no experience or special interest in mass casualty incidents expressed difficulties in coming to terms with what was going on. It is also a prominent feature from both radio communications and interviews that unexperienced personnel had more questions about their own safety and were more skeptical of entering the affected area.

An emergency response is a dynamic event, circumstances change over time. In a bomb explosion, most casualties are injured immediately and the number of casualties on scene reduced as patients is transported to health-care facilities. The amount of available resources builds up and is then reduced. The dynamics of the event can be described by dividing the response in stages. Each stage has prominent features that are recognizable across different events.

**Figure 2:**
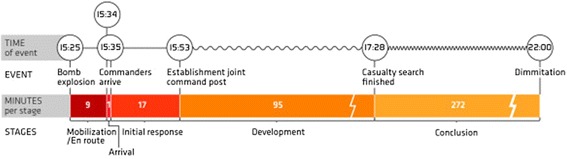
**Stages in the emergency response.** The emergency response is divided in sequential stages. The transition between stages has been defined as the time of critical events in the response operation. The timeline indicates the duration of each stage. The mobilization/en route stage started when the explosion occurred and ended when the ambulance and medical commanders parked their vehicles at the incident ground (1525 to 1534/0 to 9 min, 9 min in total). On arrival was constituted of the first minute on scene (1534 to 1535/9 to 10 min, 1 min in total). The establishment of a joint command post with police, fire, and health commanders defined the transition between initial response (1535 to 1553/10 to 28 min, 18 min in total) and development. The development stage ended when all affected buildings had been searched for casualties (1553 to 1728/28 to 123 min, 95 min in total). The conclusion stage was protracted because secondary search of the buildings needed emergency medical service presence (1728-ca 2200/123 to 395 min, 272 min in total). It ended with the last ambulance leaving the scene.

The main activity in the mobilization/en route stage is to dispatch and transport the first resources to the incident ground, cf. Figure 2. ‘On arrival’ is more of a time stamp than a stage. Direct contact with the incident will give valuable clues to confirm or rebuild the situational awareness. The initial response stage is a period of lack of resources and prioritization between lifesaving and information gathering activities. Development is a stage of better balance between needs and available resources and will typically be the stage where the bulk of patients receive treatment and are transported off the incident ground. The conclusion stage is a period of normalization of organization, discharge of resources, and little clinical activity.

### Uncertainties

**Figure 3:**
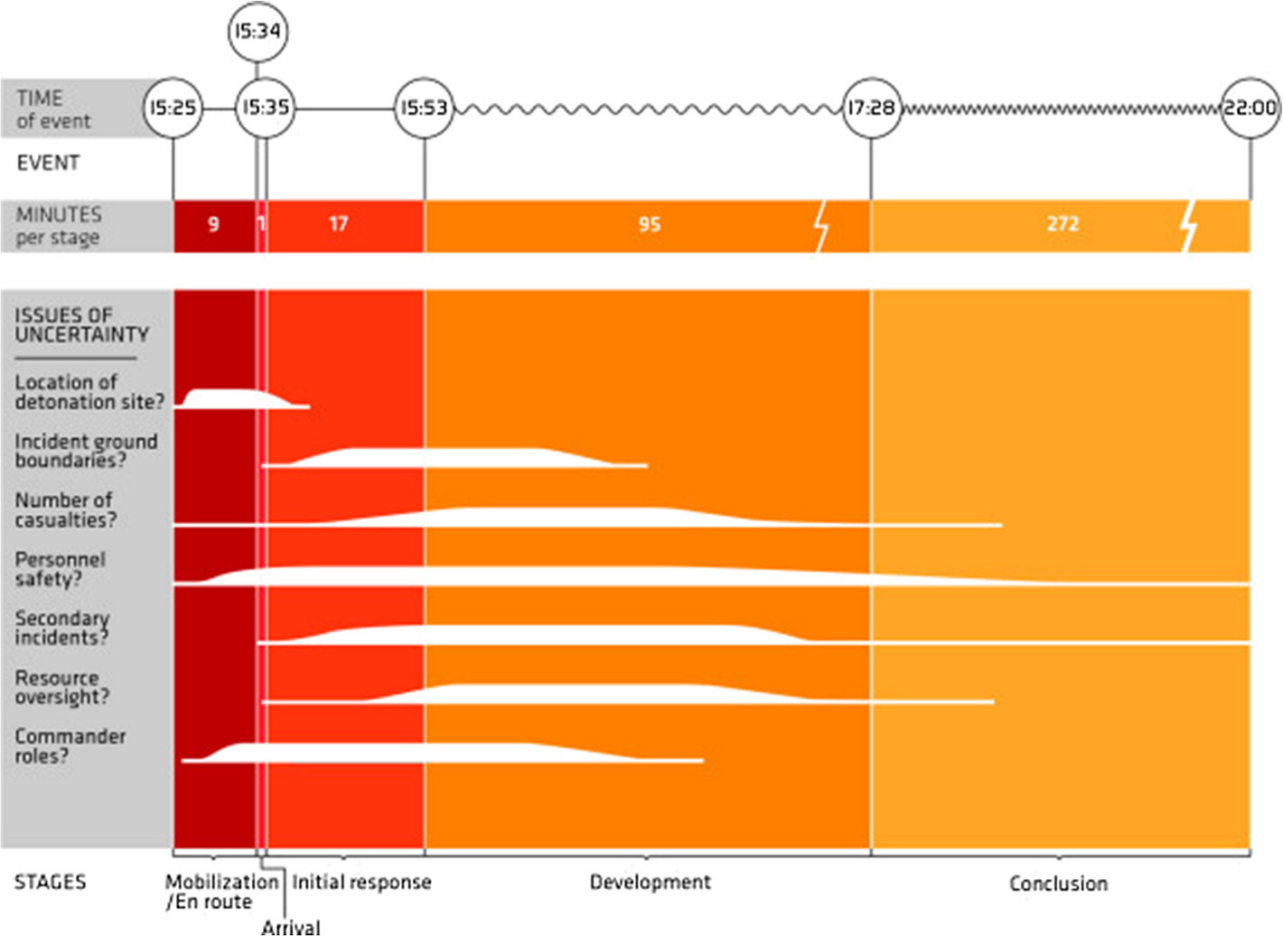
**Uncertainties.** The major issues of uncertainty are listed, and their duration and magnitude are illustrated in relation to the stages in the emergency response. The illustration represents the researchers’ interpretation of the importance of each uncertainty throughout the operation. The informants were not asked to quantify this.

A major obstacle to decision-making is the uncertainties in the factual basis for decisions. Incident commanders seldom possess a clear picture of all that is going on, and it is not clear what the consequences will be from choosing one action over another. The magnitude and importance of different uncertainties change over time, cf. Figure 3.

Conflicting information from numerous callers to the dispatch center resulted in initial uncertainty about the actual location of the detonation site. The correct location was not clear until the ambulance and medical commanders reached the site on foot a few minutes into the initial response stage.

The extent of building damage over several quarters made the boundaries of the incident ground uncertain and somewhat time consuming to establish. Clarification of boundaries was important to be able to distribute resources sensibly.

A vital information to scale the mobilization of ambulance resources is the number of casualties and the severity of their injuries. This was uncertain from mobilization throughout the initial response stage. In the development stage, the number of available resources rose and remarkably, few new patients were found. This indicated that the up till then expected large number of severely injured patients inside the buildings had been overestimated. A clear and detailed picture of the magnitude of casualties and injuries was not reached until the end of the development stage.

Personnel safety was a continuing uncertainty for the duration of the emergency response. At first, it was unclear what had happened and what safety issues might be present. After establishing the event as a bomb explosion, the main safety threats were considered to be structural damage to buildings and the possibility of a secondary explosive device.

The extensive damages and the location of the detonation site next to the building of the prime minister’s office, combined with the lack of natural explanations to an explosion in the area, immediately gave the commanders an understanding of the event as a terrorist attack. Knowledge of the *modus operandi* in previous terrorist attacks in London, Madrid, and New York made the possibility of more than one attack site a continuous uncertainty. There was no information available on the number of attackers. After reaching the point of compensation (sufficient amount of resources available on scene), this uncertainty was reduced by a mental preparedness to redirect staged resources to any new incidents.

The fast growing number of dispatched ambulances, physician staffed response cars, and depot trucks made it difficult to maintain oversight of resource distribution in the initial response stage. Debris in the streets and the mediated principle of keeping out of direct site of the detonation site if possible, both contributed to an initial scattering of vehicles. The choice of different entering points gave casualties outside the buildings treatment early, while complicating holding track of resource allocation.

### Critical decisions

**Figure 4:**
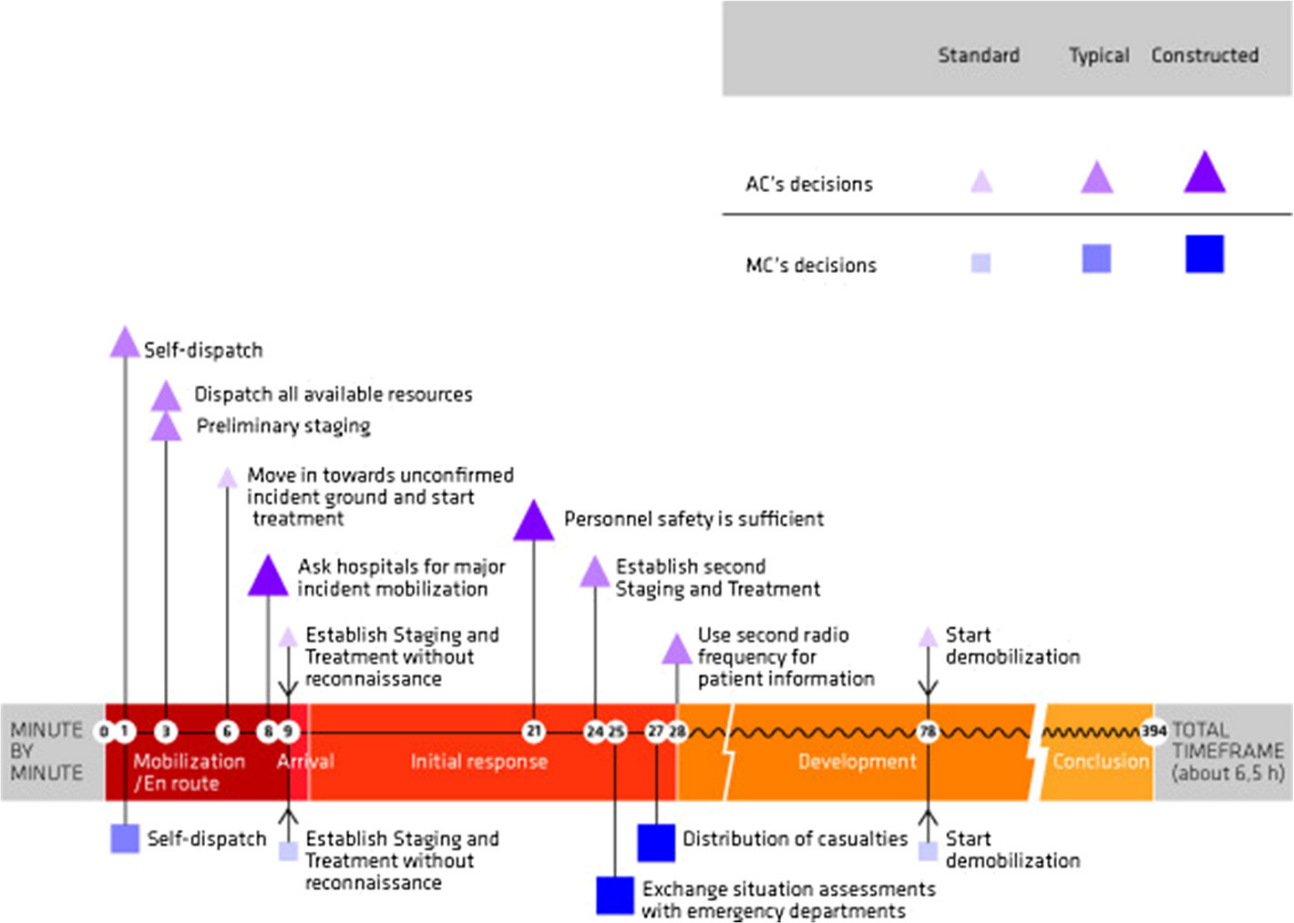
**Critical decisions.** Critical decisions made by the ambulance commander (AC) are shown above the timeline; those made by the medical commander (MC) are shown below. The decisions are categorized as standard (common in everyday work), typical (modifications to standard operating knowledge), or constructed (creative problem solving), as indicated by the size and color of the symbols [7]. Decisions marked by arrows (at 9 and 78 min) are joint decisions made after discussion between the two commanders.

A number of decisions were identified from radio communications and interviews. Only those assessed to make a significant impact on the course of the emergency response operation were categorized as critical decisions and analyzed further, cf. Figure 4.

Both the ambulance and medical commanders self-dispatched based on the initial reports of an explosion and were subsequently alarmed by the dispatch center.

The ambulance commander instructed the dispatch center to dispatch all available ambulances and stage them temporarily at the nearest ambulance station until the exact location of the incident had been established. After seeing several minor casualties in the streets, he soon decided to send the available resources into the area and start treating patients as they were encountered.

As a response to the widespread damages, but before seeing actual patients, the ambulance commander instructed the dispatch center to ask nearby hospitals to mobilize. This is normally not his call, as the decision lies with the hospitals themselves.

The ambulance and medical commanders arrived at the same time on the incident ground. On suggestion by the medical commander’s co-working paramedic, they established staging and treatment areas and appointed the paramedic staging and treatment officer.

The police asked emergency medical service personnel to stay away from the immediate surroundings of the most affected building to stay secure from a suspected second explosion. The ambulance commander decided that staging and treatment had been established on a secure spot and confirmed that work there should continue undisrupted.

The ambulance commander then established a second staging and treatment area to give better access to the most affected buildings.

The medical commander contacted by phone the emergency department at the trauma center and the nearest hospital with acute surgery functions to exchange updates on the situation and mobilization status of the hospitals. Based on this, he gave instructions on distribution of casualties between hospitals.

To reduce radio traffic, the ambulance commander decided that ambulances leaving with patients should use a second frequency to inform the dispatch center of patient details.

After 78 min, the ambulance and medical commanders decided that there were enough resources at staging. Subsequently released units were commanded by the dispatch center on a third radio frequency.

After a joint discussion between all commanders, decision was made to release most of the remaining resources. During this meeting, the commanders were notified of the ongoing shooting at Utøya Island. A considerable number of the released ambulances were then dispatched to Utøya.

## Discussion

**Figure 5:**
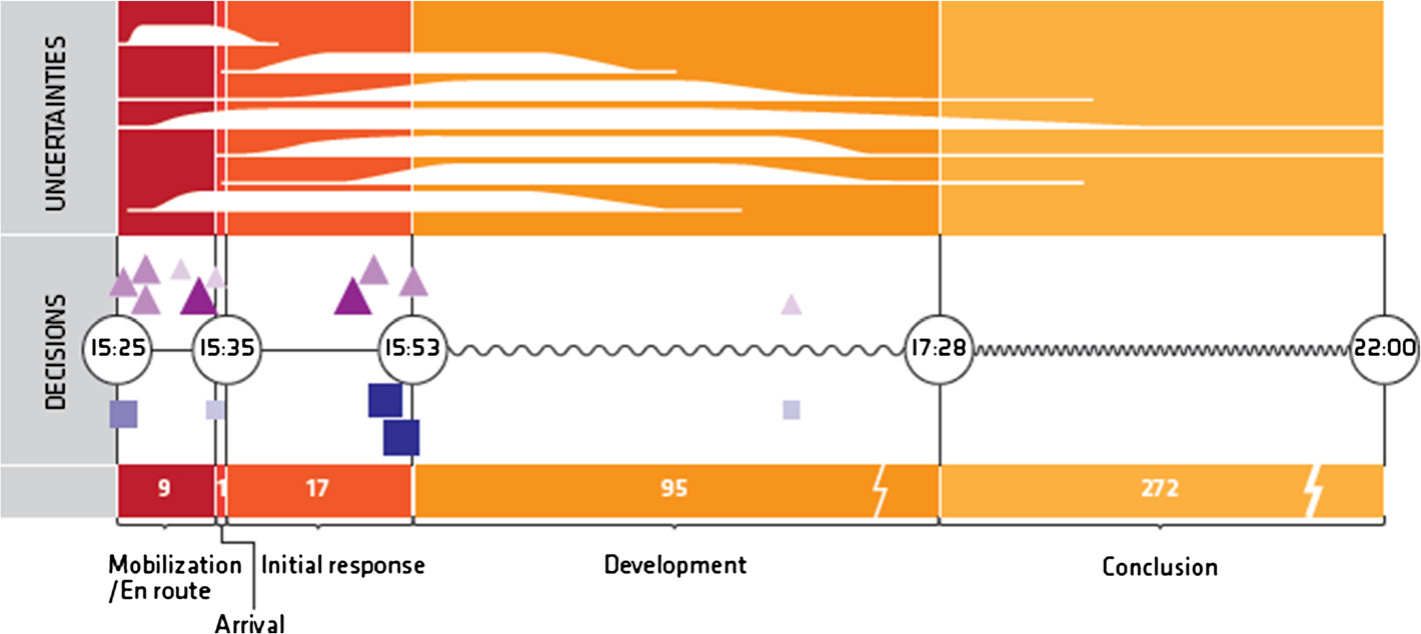
**Stages, uncertainties, and critical decisions.** Based on knowledge and experience, the commanders used visual, audio, and olfactory cues to rapidly make situation assessments. The focus of attention and the overall situational awareness changed throughout the stages of operation: mobilization/en route, on arrival, initial response, development, and conclusion. The temporal correlation of uncertainties and critical decisions is shown in relation to the different stages of the emergency response. Major uncertainties affecting decision-making included location and boundaries of the incident ground, number of casualties, commander roles, personnel safety, and lack of resource oversight. The number and magnitude of uncertainties was largest in the development stage, after most of the critical decisions had been made. Details and the use of symbols for each of these factors are presented in Figures 2, 3, and 4.

In the 2011 Oslo bombing emergency medical response operation, the ambulance commander made critical decisions regarding dispatch, distribution, and demobilization of resources, major incident mobilization in nearby hospitals, and conditions for personnel safety. The medical commander made critical decisions regarding communication with and distribution of patients to nearby health institutions, as well as distribution and demobilization of resources. The ambulance commander was highly visible as a decision maker to all personnel. The medical commander had a withdrawn, monitoring role. Most critical decisions were made within the first 30 min of the operation, cf. Figure 5.

### Command role clarity

The ambulance and medical commanders express uncertainty regarding their command roles and how these were to be conducted in the best way. This was most prominent in the early stages of the response. The basic conditions for the two commanders’ role execution were quite different. The ambulance commander has a 24/7 function, meaning that there is always an ambulance commander on call. They have ambulance command as their main duty when on call, and this is their everyday job. They are familiar and acquainted with their peers in the fire and police forces through numerous smaller incidents like road traffic accidents and building fires, and through joint training. At most of these incidents, the medical commander role is not activated. In 2011, there was a physician manned response car in Oslo at daytime Monday to Friday, and two helicopter units using response cars for missions within the city. These physicians can function as medical commander in large-scale incidents, but this is not often done and is clearly a secondary role.

The interaction between the ambulance and medical commanders was thus not based on tacit knowledge and experience from everyday work. The ambulance commander could take on his normal role in relation to the dispatch center and the ambulance personnel on scene but had to negotiate decisions with the medical commander. An additional uncertainty was the arrival of a second ambulance commander. He was appointed as a deputy ambulance commander, a less familiar role as the ambulance commanders are used to work alone.

Physicians are in general used to work in shifting teams and to be overall responsible for the treatment of single patients, which may ease the medical commander’s close interaction with the ambulance commander and other commanders or subordinates. A major obstacle was the poor definition and familiarity of the role itself. The medical commander was less prominent or visible to the on scene personnel, and most informants had a quite blurry picture of what tasks he had performed. Keeping in contact with emergency departments and providing decision support to the ambulance commander are less visible tasks.

### Situation assessment and critical decisions

The commanders’ overall assessment was that this incident was extraordinary. The amount of critical decisions by the ambulance commander during the first few minutes was affected by this awareness. The significant presence of constructed decisions also highlights that this was not an everyday emergency response.

As discussed, experience enabled personnel to use critical cues to build situational awareness based on recognition. Under the naturalistic decision-making paradigm, it can further be argued that these experienced experts are more prone to rapidly choose a workable option than to weigh all possible options thoroughly [12]. An example is how staging and treatment areas were rapidly established and subsequently moved and adjusted throughout the emergency response, as opposed to delaying the decision in search for the optimal location and thereby delaying the onset of operations.

### Situation assessment and uncertainties

Dividing the emergency response in stages may be a convenient analytic tool to researchers, but its main purpose is to help build or frame the situation assessment of personnel in the field. The ambulance commander actively used this by declaring, ‘We are now in the development stage’ in radio transmissions. This signifies a degree of oversight, established on scene organization, and current demand-resource balance, communicated easily through a short fraise.

On the other hand, there were throughout the first 2 h of the operation constantly new units being dispatched. These crews are mentally and practically in the mobilization/en route stage and will use some time on scene to develop the necessary familiarity with the situation and organization to effectively reach ‘their’ development stage. The commanders on scene enhanced their own situational awareness by actively avoiding focus on resources in other stages and different locations and ordered the dispatch center to deal with them.

Whereas the ambulance commander was quite tied up in radio communication and non-critical or more detailed decisions, the medical commander could take on a more withdrawn and monitoring position. This enabled a bird’s eye view of the situation and made it easier to assess resource balance and suggest downsizing at an earlier stage. This difference in approach and therefore situational awareness may be one of the advantages of this dual command model.

### Uncertainties and critical decisions

The size of the affected area and the presence of physical obstacles (mainly buildings) on the incident ground made visual oversight over the area and direct contact with all personnel impossible. This functioned as an incentive to divide the organization into geographical and functional units with sub-commanders, each with a more manageable area of command. Command over units not physically present on the incident ground was left to the dispatch center. These decisions contributed to protect the commanders from involvement in matters outside the geographical area and thereby reduce complexity and uncertainty.

Most of the significant uncertainties were, however, not available for reduction by decisions. The main interaction between uncertainties and decisions was that the uncertainties triggered decisions to be made without certain knowledge of what might be the best option.

The initial uncertainty regarding the location of the explosion and the extent of the incident was met by sending in the first available resources to start treatment of the known casualties, without waiting for a thorough reconnaissance before starting rescue activities. The ambulance commander made this decision with knowledge from experience that a sufficient amount of resources would be mobilized within a reasonably short time, so that strict rationing of personnel was not of the essence. A less experienced commander might have chosen a more careful approach and held back vehicles at staging to be sure to remain in detailed control when sending the ambulances into action. In this case, the experienced commander handled the added uncertainty of scattering resources.

Uncertainties concerning personnel safety lead to the decision to place resources where they would be protected from a secondary explosion or building collapse. An even more cautious approach would have been to keep resources out of the area entirely until definitive security clearance from the police and fire departments. The commanders mutually agreed that this was unnecessary and rather focused on keeping the on scene time low for both casualties and personnel, as advised by Aylwin and colleagues [21].

The ambulance and medical commanders stayed close together on arrival and in the initial response stage. This made negotiations on tasks and responsibilities swifter. At the later stages, there was less uncertainty as to who should make decisions on what, and the commanders split up for longer periods of time.

Perceived danger of multiple attack sites was a driver towards staging excessive resources on scene and mentally preparing to expand or split the organization to cover new incident grounds. The decision was later made to discharge personnel and return them to their home bases to restore the general preparedness.

### Limitations

A recall bias was introduced by the time lag between the incident and the interviews. Radio communication records are not subject of the same bias, but it is not the only way of communication in an emergency response operation, and significant data may have been missed if not captured in the interviews.

Systematic text condensation is inspired by Giorgi’s phenomenological analysis and resembles grounded theory. The analysis method is well suited for descriptive studies based on information from numerous informants or sources. Preliminary analysis during data collection, a stepwise analysis process with development of the coding tree, and reformulation of research questions as part of the analysis are recommended features of the method.

A crucial methodological point is that, in this study, one of the researchers analyzes his own decisions and actions. This may obviously be seen as a weakness. On the other hand, participating observation is a good method for developing process-oriented explanations, and it is highly recommended for the researcher to actively use his own experiences and involvements [22,23]. The research questions are more focused on what decisions were made than on how they were made or whether the decisions and the overall conduct of command were of a good or bad quality. The study is as such not an evaluation of performance, and the first-hand knowledge of context and familiarity with the data material may be seen as a strength to this descriptive observational study.

The framework for separating the timeline in stages used here was developed from experience by British fire and rescue officer. There is no official standard in Norway, nor are we aware of any international consensus on definitions and naming of stages in a mass casualty incident. We found this framework intuitive, usable, and well documented.

### Further research

A qualitative analysis based on data from a single case is not generalizable, and findings need to be validated and developed through further research conducting similar analyses of critical decisions in other mass casualty incidents.

## Conclusions

Making critical decisions fast on the basis of pattern recognition and then adjust later, as situation assessment requires, is superior to omitting or delaying decisions in order to eliminate uncertainties in a mass casualty incident. Uncertainties will be present when critical decisions are made. Preparing potential ambulance and medical commanders in situation assessment and decision-making in the presence of uncertainty may prove a valuable addition to practical training in incident management.

## Additional file

Additional file 1:
**Interview guide.** Results of the preliminary analysis of radio communication transcripts were used as an interview guide in semi-structured interviews.
