# Supplementary material for: A retrospective observational study of medical incident command and decision-making in the 2011 Oslo bombing
Source: Int J Emerg Med. 2015 Mar 4;8:4. doi: 10.1186/s12245-015-0052-9 (PMC4385130; doi:10.1186/s12245-015-0052-9)
Supplement: Additional file 1: — Interview guide. Results of the preliminary analysis of radio communication transcripts were used as an interview guide in semi-structured interviews. [file 12245_2015_52_MOESM1_ESM.pdf]

Interview guide

Establish an incident timeline for the informant

-narrative

-critical moments or turns of events

Situational assessment

-changes

-cues

-previous experience

Anticipated development of events

-others’

-own

-changes in anticipation

Distribution of resources

-frame or directions

-explicit orders

-own initiative

Tasks

-frame or directions

-explicit orders

-own initiative

Organizational structure

-frame or directions

-explicit orders

-own initiative
